# Towards the characterisation of sustainable diet’s gut microbiota composition and functions: A narrative review

**DOI:** 10.1017/gmb.2023.13

**Published:** 2023-09-11

**Authors:** Mariana Lares-Michel, Zyanya Reyes-Castillo, Fatima Ezzahra Housni

**Affiliations:** 1 Institute of Nutrition and Food Technology “José Mataix Verdú”, Biomedical Research Center, University of Granada, Avenida del Conocimiento S/N, Parque Tecnológico de la Salud, Armilla, 18071 Granada, Spain; 2 Instituto de Investigaciones en Comportamiento Alimentario y Nutrición (IICAN), Centro Universitario del Sur, Universidad de Guadalajara, Av. Enrique Arreola Silva 883, Col. Centro. 49000, Cd. Guzmán, Jalisco, México

**Keywords:** sustainable diets, gut microbiota, gut microbiome, plant-based diets, planetary health

## Abstract

The gut microbiome is a key element for health preservation and disease prevention. Nevertheless, defining a healthy gut microbiome is complex since it is modulated by several factors, such as host genetics, sex, age, geographical zone, drug use, and, especially, diet. Although a healthy diet has proven to increase microbial alpha and beta diversity and to promote the proliferation of health-related bacteria, considering the current environmental and nutritional crisis, such as climate change, water shortage, loss of diversity, and the obesity pandemic, it should be highlighted that a healthy diet is not always sustainable. Sustainable diets are dietary patterns that promote all dimensions of people’s health and well-being while exerting low pressure on the environment, and being accessible, affordable, safe, equitable, and culturally acceptable. Examples of diets that tend to be sustainable are the Planetary Health Diet of the EAT-Lancet Commission or territorial diets such as the Mediterranean and the Traditional Mexican diet (milpa diet), adapted to specific contexts. These diets are principally plant-based but include small or moderate amounts of animal-based foods. Characterising the effects of sustainable diets on gut microbiota is urgent to ensure that the benefits for human health are aligned with environmental preservation and respect the sociocultural aspects of individuals.

## Graphical abstract

**Figure:**
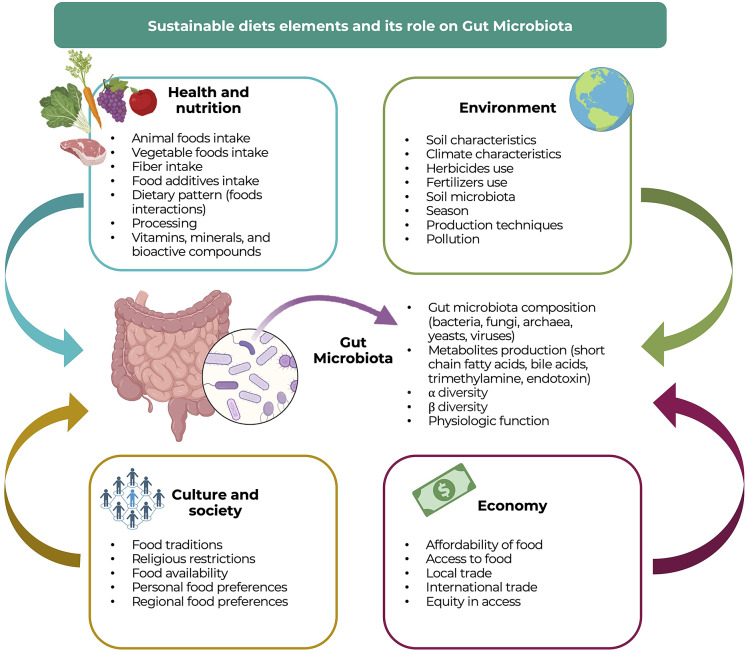

## Introduction

Currently, we know the gut microbiome is one of the most critical determinants of health and its alterations are associated with the development of chronic degenerative diseases (Vijay and Valdes, 2022). It also regulates several immunological, metabolic, physiological, and structural functions and even has a role in behaviour (Johnson and Foster, 2018; Vijay and Valdes, 2022). The gut microbiome is modulated by several factors, such as genetics, sex, age, geographical zone, drug use, and, especially, diet. The understanding of the role of diet in the composition and functions of gut microbiota has increased significantly in the past decade (Rinninella et al., 2023). Growing research indicates that a healthy diet could be the key element of a “healthy” gut microbiome, which is composed of bacteria generally associated with metabolic health and with little or no pathogenic bacteria. However, it is essential to mention that a healthy gut microbiome can vary significantly across hosts since each human can respond differently to the same nutrients, and the same bacteria can have different effects on each individual (Rinninella et al., 2023; Shanahan et al., 2021). Besides, the gut microbiome can be a fingerprinting of each individual or even of specific disease-related bacteria (Tierney et al., 2019).

Although some aspects of gut microbiota are consistent through literature, such as the fact that plant-based and high-fibre diets are related to bacteria such as *Faecalibacterium prausnitzii*, which is associated with anti-inflammatory effects (Sidhu et al., 2023; Trefflich et al., 2020), whereas Western diets based on ultra-processed foods are related to dysbiosis (Rinninella et al., 2019), these trends in research have spread and reinforced the idea that a healthy diet could be the key to having a gut microbiome as healthy as possible. However, when addressing diets in the context of the current environmental crisis it is essential to remember that their impact is not limited to human health, but also their production has critical environmental impacts. In this sense, recent research has evidenced that a healthy diet is not necessarily sustainable (Birney et al., 2017; Macdiarmid, 2013).

The sustainable diets concept is complex and integrates four essential dimensions: (1) health and nutrition, (2) environment, (3) economy, and (4) culture and society (Auestad and Fulgoni, 2015). The most up-to-date definition of sustainable diets was provided by the Food and Agriculture Organization of the United Nations and World Health Organization (2019), which defines it as “dietary patterns that promote all the dimensions of people’s health and well-being; they have low pressure and environmental impact; they are accessible, affordable, safe and equitable; and are culturally acceptable” (p. 9).

Some of the main characteristics of sustainable diets are that they are locally produced, with little or no processing, and mainly of vegetable origin. However, as the concept itself indicates, multiple factors are directly and indirectly related, for example, the characteristics of where the diet is produced. In the sustainability context, biodiversity is essential to produce dense nutrient foods, and some regions are more productive than others. Several aspects are implicated in soil fertility, among which soil microbial diversity is essential. In this regard, McCarthy and Li (2019) were the first authors to point out the importance that some promoters of sustainable diets, such as the EAT-Lancet Commission, start considering not only preserving species diversity in the earth’s biosphere but also promoting diversity of microbial species in the human gut. In this sense, although the characterisation of the gut microbiota in relation to plant-based diets, such as vegan, vegetarian, and Mediterranean, is currently a field of intense research, the specific effects that specific sustainable diets, locally produced, have on specific human populations have not been reported. Therefore, this review explores the literature regarding potential sustainable diets’ effects on gut microbiota composition and functions.

## Healthy diet’s composition and its effects on the gut microbiota … are they sustainable?

A sustainable diet is a relatively new concept that aims to integrate health and nutrition with the environment and socio-economical aspects of the population to preserve the planet (Food and Agriculture Organization of the United Nations and World Health Organization, 2019). Although sustainable diets must be contextualised to be considered sustainable, in the current literature it is common to assume healthy diets as sustainable (Sidhu et al., 2023). A healthy diet is defined as a health-promoting and disease-preventing diet. It provides adequacy, without excess, of nutrients and health-promoting substances from nutritious foods and avoids the consumption of health-harming substances (Neufeld et al., 2021).

Healthy diets are generally assumed as sustainable, and although in some cases it is true, this cannot be generalised (Lares-Michel et al., 2021). Indeed, some studies have found that shifting current dietary patterns to dietary guideline recommendations would increase dietary environmental impact in several ways; for example, an increase of 15% in blue water footprint, 34% in energy use, 7% in dietary greenhouse gas emissions, and 34% in fertiliser use (Birney et al., 2017).

Dietary guidelines are consistent worldwide, recommending a high consumption of vegetables, fruits, and whole grains, which must constitute half of a person’s daily diet. Besides, all dietary guidelines agree on moderating and/or avoiding refined grains, sugar, saturated fats, and ultra-processed foods (Armet et al., 2022). Regarding animal-based foods, recommendations vary a little more. Generally, all dietary guidelines promote moderation in meat, dairy, and egg consumption (Armet et al., 2022). Recently, some guidelines recommend substituting meats for legumes, nuts, and seeds (Cena and Calder, 2020; Food and Agriculture Organization of the United Nations, 2023). However, country-specific dietary guidelines have not distinguished recommendations regarding critical elements in healthy sustainable diets, for example, the environmental impact of specific foods, especially meats (i.e., beef, pork, chicken, goat, and fish) (Cena and Calder, 2020; Food and Agriculture Organization of the United Nations, 2023). Most dietary guidelines group red meat, poultry, fish, processed meats, and even legumes (Macedo-Ojeda et al., 2016). Besides having completely different environmental impacts, their effects on the gut microbiota and their related metabolites vary greatly (Lonnie et al., 2018; Świątecka et al., 2011).

From an environmental sustainability perspective, beef’s international average water footprint is 15,415 litres per kilogram (L/kg) (Mekonnen and Hoekstra, 2010); meanwhile, this figure can rise to more than 21,000 L/kg in water-scarce regions such as Mexico (Hoekstra et al., 2012). Other meats, such as chicken, can have considerably lower impacts. For example, its water footprint is 4,325 L/kg, and pork has a water footprint of 5,988 L/kg. Similarly, greenhouse gas emissions vary greatly among meat types. Beef, for example, reaches more than 34 CO_2_eq/kg, while the carbon footprint of chicken is 8.58 CO_2_eq/kg and of fish is 11.62 CO_2_eq/kg (López-Olmedo et al., 2022). Besides, most dietary guidelines widely recommend the intake of nuts as a protein and/or healthy fats source (Armet et al., 2022). However, from an environmental perspective, its impact is even higher than most meats, with a water footprint of 9,063 L/kg (Mekonnen and Hoekstra, 2012). The same occurs with olive oil, which has a water footprint of 14,431 L/kg (Mekonnen and Hoekstra, 2010). Nevertheless, its carbon footprint is lower (3.10 CO2eq/kg) (Grasso et al., 2020), and it should be considered that healthy fats can be included in a healthy sustainable diet in adequate portions, since a tablespoon of olive oil has only a water footprint of 216 litres. Meanwhile, servings of beef tend to be higher, for example, over 100 g, which would have a water footprint of 1,541 litres (Mekonnen and Hoekstra, 2010). This highlights the importance of intake of recommended consumption amounts in current dietary guidelines.

Regarding gut microbiome, it is interesting that the variations found in environmental sustainability tend to align with the type of bacteria associated with each food group and specific metabolites. For example, the foods with lower environmental impact, which are vegetables, fruits, whole grains, and legumes (Willett et al., 2019), are the leading food groups associated with anti-inflammatory bacteria such as *F. prausnitzii*, *Prevotella copri,* and *Akkermansia muciniphila* (Avila-Nava et al., 2017; Sidhu et al., 2023; Trefflich et al., 2020). However, this is not a rule since although nuts and olive oil have proved to increase the proliferation of metabolic health-associated bacteria, such as *Bifidobacterium spp.*, and reduce pro-inflammatory bacteria, such as Pseudomonadota (previously called Proteobacteria) (Marcelino et al., 2019), their impact on water use generates controversy about the amounts recommended in current healthy dietary guidelines (Blas et al., 2016).

The same occurs with avocado, which has been widely recognised as essential to maintaining a diverse gut microbiome (increasing alpha diversity) and enriching bacteria such as *Faecalibacterium*, *Lachnospira*, and *Alistipes.* Besides, avocado consumption, in quantities from 175 g in men to 140 g in women, also modifies the human microbial metabolites (Thompson et al., 2021). A randomised controlled trial (RCT) found increases in faecal acetate, stearic acid, and palmitic acid concentrations. In contrast, the concentrations of the bile acids, cholic and chenodeoxycholic acid, were 91% and 57% lower in persons consuming avocado compared to a control group. Also, its consumption favours greater fecal short-chain fatty acids (SCFAs) concentrations (Thompson et al., 2021).

Nevertheless, avocados’ production leads to critical environmental impacts (De la Vega-Rivera and Merino-Pérez, 2021). Although the avocado crop itself has a relatively low environmental impact, with around 900 L/kg of water footprint (Mekonnen and Hoekstra, 2010) and 1.34 CO_2_eq/kg (López-Olmedo et al., 2022), the agribusiness dedicated to massive exports in countries like Mexico has brought severe environmental consequences (De la Vega-Rivera and Merino-Pérez, 2021; Macias, 2015).

Another food group that worries sustainability experts is dairy. Dairy is included in most dietary guidelines (Armet et al., 2022) since these are dense nutrient foods that also benefits the gut microbiota by increasing beneficial genera *Lactobacillus* and *Bifidobacterium*, and reducing pathogenic bacteria such as *Bacteroides fragilis* (Aslam et al., 2020). However, its role in sustainability remains controversial since it is considered one of the food groups with the highest environmental impact (Mekonnen and Hoekstra, 2012; van Hooijdonk and Hettinga, 2015). Nevertheless, the caloric and fat intake rise significantly when substituting dairy for plant-based foods to obtain nutrients such as calcium (i.e., by consuming almonds) (Rodríguez Huertas et al., 2019). Besides, these changes would also lead to even higher environmental impacts since, although plant-based, some dairy substitutes such as almond drinks have a higher environmental impact, especially from a water use perspective (Carlsson Kanyama et al., 2021).

Nowadays, other dietary patterns that can be considered healthy are high protein diets, which have gained attention in the last years, especially for losing weight (Moon and Koh, 2020) or regulating metabolic diseases such as type 2 diabetes (Malaeb et al., 2019). However, their effects are still inconclusive (Malaeb et al., 2019). With regard to the gut microbiota, the effect of high protein diets has been widely studied, showing that high animal protein intake leads to higher Pseudomonadota such as *Deferribacteres* (Senghor et al., 2018) and decreases *Bifidobacteria* (Rinninella et al., 2019). Also, high animal protein consumption promotes higher proliferation of the pathobionts *Bilophila* and *Lachnoclostridium* (Wu et al., 2022), as well as *Bilophila wadsworthia*, which is also related to pro-inflammatory effects and the development of chronic and gastrointestinal diseases (David et al., 2014).

Besides the environmental impact, most dietary guidelines do not consider food costs (Food and Agriculture Organization of the United Nations, 2023). This has been repeatedly referred to as one of the main barriers to adopting a healthy diet, and, currently, a healthy and sustainable diet (Barosh et al., 2014). This could also be directly linked to dysbiosis by preferring cheap food that tends to be fast foods or ultra-processed foods, which have been proven to alter the gut microbiota, generating dysbiosis (Aguayo-Patrón and de la Barca, 2017). Besides, a cross-sectional study found interesting alterations in the gut microbiota of women and men consuming ultra-processed foods. It was found that women consuming more than five servings per day of these foods had more bacteria such as *Acidaminococcus*, *Butyrivibrio*, *Gemmiger*, *Shigella*, *Anaerofilum*, *Parabacteroides*, *Bifidobacterium*, *Enterobacteriales*, *Bifidobacteriales*, and Actinomycetota (previously called *Actinobacteria*), and a decrease in *Melainabacter* and *Lachnospira.* Meanwhile, men who consumed the same amount of ultra-processed foods presented an increase of *Granulicatella*, *Blautia*, *Carnobacteriaceae*, *Bacteroidaceae*, *Peptostreptococcaceae*, *Bacteroidia,* and Bacteroidota, and a decrease of *Anaerostipes* and *Clostridiaceae* (Cuevas-Sierra et al., 2021).

Finally, although each dietary guideline is country-specific, cultural and social elements are not always included, especially at the individual level (Food and Agriculture Organization of the United Nations, 2023). Among cultural aspects, cooking and region-specific recipes can play an essential role in the gut microbiome (Carmody et al., 2019). Heat alters the physicochemical properties of foods in ways that could impact the gut microbiome. Cooking increases the ileal digestibility of carbohydrates by gelatinising starch, reducing the quantity reaching the colon, where the most numerous microbial community resides, and potentially affecting the fermentation capability of amylolytic gut bacteria. Cooking can also denature antimicrobial compounds present naturally in food or introduced through agriculture, thus limiting their bioactivity (Carmody et al., 2019).

Specifically, a study showed that cooking methods and ingredients for cooking can alter gut microbiota composition. For example, it was shown that butter positively impacts the abundance of potentially advantageous taxa, including *Faecalibacterium*, *Roseburia*, and *Blautia.* However, butter and other high-fat animal foods, such as dairy products and fish, also resulted in higher abundances of *Lachnoclostridium*, which has been associated with several diseases. Frying was identified as the cooking method producing the most distinct effects on the microbiota when contrasted to other methods of cooking a particular food. This could be related to its high generation of Maillard reaction products. However, in general, the impact of cooking methods on the effects of foods was highly varied across individuals (Lerma-Aguilera et al., 2022). This confirms the importance of personalised and precision nutrition. Besides, behavioural elements of individuals must be considered in healthy diet promotion to be considered sustainable dietary patterns (Lares-Michel et al., 2023).

## Plant-based diets

Animal-based foods have been widely recognised as having the highest environmental impact (Willett et al., 2019). Among those, meats, especially beef, are considered the food with the highest impact from all environmental sustainability perspectives, being the main contributors to climate change because they have the highest greenhouse gas emissions and land and water use (Scarborough et al., 2014; Willett et al., 2019). For this reason, dietary patterns restringing animal foods, such as plant-based diets, are constantly considered sustainable (Carey et al., 2023). According to a recent systematic review, plant-based diets generate lower greenhouse gas emissions (GHGEs), use less land, and limit biodiversity loss than standard diets. Nevertheless, the impact on water and energy use may depend on the types of plant-based foods consumed. However, the paper concludes that, in general, plant-based dietary patterns reduce diet-related mortality and also promote environmental sustainability (Carey et al., 2023).

Some examples of plant-based diets are vegan, vegetarian, pescatarian, and flexitarian diets (Sidhu et al., 2023). Vegan diets are plant-based and omit all animal products, even eggs, dairy products, and honey (Losno et al., 2021; Melina et al., 2016). A vegetarian diet is a plant-based diet that may or may not include egg or dairy products (Melina et al., 2016). Pescatarians are defined as vegetarians who consume fish and seafood (Wozniak et al., 2020). Finally, flexitarian or semi-vegetarian diets are a recent concept that refers to a plant-based dietary pattern with occasional beef, pork, poultry, or fish, perhaps once or twice weekly (Melina et al., 2016).

Although, as said before, plant-based diets are generally aligned with environmental sustainability, health, nutrition, socioeconomic and cultural aspects are not always respected in this kind of diets. Besides, specific elements of these diets’ environmental impact are not always considered. Additionally, cultural and socioeconomic elements of plant-based diets are very controversial since they tend to be very distant from the traditional and regional diets of current populations, in addition to the fact that they do not usually align with the food preferences of the general population, besides being considered expensive diets for some specific socioeconomic strata (Drewnowski, 2020; Goulding et al., 2020). Despite this, plant-based diets’ effects on the gut microbiome have been called attention to in the last decade because those diets have a high content of microbiota-accessible carbohydrates (MACs) that are not digested and absorbed by the host, such as resistant starch, inulin, xylan, and pectin. These compounds favour the growth of specialised taxa like *Ruminococcus bromii*, *Roseburia intestinalis*, and, especially, *F. prausnitzii*, which is associated with anti-inflammatory effects (Shetty et al., 2022; Sidhu et al., 2023; Trefflich et al., 2020). Also, high-fibre diets rich in MACs are reported to benefit host physiology via enhanced butyrate production and promote higher bacterial diversity (Shetty et al., 2022). According to Losno et al. (2021), the genus that is consistently reported as higher in vegans in contrast with omnivores is *Prevotella.* Specifically, *P. copri* has been reported to increase in vegans compared to omnivores (Trefflich et al., 2020).

Among plant-based diets, those that include some animal-based foods, such as pescatarians, have been shown to promote different gut microbiomes in the host, contrasting dietary patterns without animal foods. However, compared to the omnivore and vegetarian groups, a significantly higher Bacillota/Bacteroidota ratio (initially named as Firmicutes/Bacteroidetes ratio) has been reported in the pescatarian and vegan groups (Shetty et al., 2022). This could indicate that some shared components in those diets, such as dairy and eggs in omnivores and vegetarians, could be the main determinants of gut microbiota distinctions. Meanwhile, shared high fruit and vegetable intake in vegans and pescatarians could modulate similar ratios between Bacillota (previously called Firmicutes) and Bacteroidota (previously called Bacteroidetes) (Shetty et al., 2022). However, fish intake plays an inconclusive role on the gut microbiome because although its regular consumption lowers rates of ischemic heart disease than meat eaters, fish intake generates significant increases in plasma circulating levels of Trimethylamine-N-oxide (TMAO), which rise 15 min after a meal, reaching approximately 50 times higher circulating concentrations of TMAO than beef or egg consumption (Cho et al., 2017; Landberg and Hanhineva, 2019).

From an environmental, economic, and social perspective, fish consumption is controversial since it can become an expensive source of protein and omega-3 fatty acids (Batis et al., 2022) and is not linked with all traditional dietary patterns worldwide. For example, Mediterranean countries consume considerably more fish than other regions, such as India (Food and Agriculture Organization of the United Nations, 2020).

## Territorial diets: Mediterranean and Nordic diets

Territorial diets (i.e., regionals) have stood out as sustainable dietary patterns among healthy diets. Among those, the Mediterranean diet is one of the most recognised worldwide as a healthy dietary pattern, and its beneficial effects on the gut microbiome are well-established (Bach-Faig et al., 2011; Meslier et al., 2020). The Mediterranean diet comprises vegetables, fruits, legumes, nuts, olive oil, and fish; additionally, it includes low amounts of red meat, dairy products, and saturated fats (Ghosh et al., 2020; Serra-Majem et al., 2020). Multiple RCTs have evidence that regular consumption promotes the proliferation of health-related bacteria. In an RCT conducted on individuals with excess body weight, increased adherence to a Mediterranean diet decreased plasma cholesterol concentrations and enriched *F. prausnitzii* and *Roseburia* abundances compared with a control diet (Meslier et al., 2020). These bacteria were also identified as good predictors of dietary adherence scores in an RCT in older adults who consumed a Mediterranean diet for 12 months (Ghosh et al., 2020).

Another study based on an intervention promoting adherence to the Mediterranean diet identified a decrease in *Butyricicoccus*, *Haemophilus*, *Ruminiclostridium*, and *Eubacterium hallii* in the participants consuming that diet compared with a control group. Also, changes in *Lachnospiraceae NK4A136* were positively associated with changes in adherence to the Mediterranean diet (Muralidharan et al., 2021). Similarly, specific taxa such as *Anaerostipes hadrus* showed high abundances in a Mediterranean diet enriched with fibre. Besides, *Agathobaculum* and *Anaerostipes* genus and *Agathobaculum butyriciproducens* and *Anaerostipes hadrus* species showed significantly higher (*p* < 0.05) abundances after an intervention with the Mediterranean diet. However, since only changes in specific taxa were observed, no significant effects on the core microbiota could be reported (Barber et al., 2021).

Although the Mediterranean diet is commonly assumed as a sustainable dietary pattern (Fresán et al., 2018; Hachem et al., 2020; Serra-Majem et al., 2020), some studies analysing their environmental impact and affordability have found diverse results that indicate that although being sustainable in some aspects, such as GHGE and affordability in some socioeconomic strata (Germani et al., 2014), other indicators, such as the water footprint, tend to present high environmental impacts (Blas et al., 2016). Also, it is not affordable for some population sectors (Rubini et al., 2022). This emphasises the importance of contextualising and individualising diets to be considered sustainable and, thus, to characterise their specific effects on the gut microbiome.

Another diet recognised as healthy and, recently, as sustainable is the New Nordic diet (Hachem et al., 2020; Landberg and Hanhineva, 2019). It is characterised by high content of local vegetables such as cabbages, mushrooms, root vegetables, and fruits such as berries, apples, and pears. It includes legumes, fresh herbs, potatoes, whole grains (Barley, rye, oats, spelt, and buckwheat), native nuts (hazelnuts, walnuts, and chestnuts), fish and shellfish, seaweed, and free-range livestock like pigs and poultry (Meltzer et al., 2019; Mithril et al., 2013). It also comprises traditional foods sourced in the Nordic countries and focuses on those from the wild countryside and the sea and lakes (Hachem et al., 2020). Various studies have shown their beneficial effects on the gut microbiome. For example, interventions with whole grains have increased SCFA-producing *Lachnospira spp.* compared with a control group consuming refined grains (Vanegas et al., 2017).

Despite its effects, a study showed that a dietary intervention with the New Nordic diet in adults 18-65 years did not affect the ratio when the abundances of 35 selected bacterial taxa were quantified before and after the intervention. Also, the study showed that the participant’s enterotype appeared to significantly impact the total plasma cholesterol more than diet. Besides, higher levels of the *Prevotella*/*Bacteroides* ratio were associated with higher total cholesterol levels (Roager et al., 2014). Other studies assessing the effects of diets with the New Nordic Diet characteristics, such as whole grain diets, have only shown minor alterations in the gut microbiota. However, this diet has been associated with increased SCFA-producing *Lachnospira spp.* when compared with a control group consuming refined grains (Landberg and Hanhineva, 2019).

Despite the well-demonstrated benefits of this diet on the gut microbiome, it is essential to mention that, from a sustainability perspective, there are elements that have not been addressed, such as the environmental impact of the persons following the dietary interventions previously mentioned. Besides, it is not mentioned if all participants had access to the same type and quality foods in the diet. Actually, a study mentioned that typical current diets in the Nordic countries are neither healthy nor environmentally sustainable, although there are indications of progress towards more fruit and vegetables in the diet. This could lead to essential differences in health outcomes, especially in the gut microbiome (Meltzer et al., 2019).

## Traditional diets: The Milpa diet

The European colonisation of American countries modified the traditional and Indigenous diets of populations such as the Mesoamericans (Palka, 2009). Among those is the Mexican traditional pre-Hispanic diet, also known as the milpa diet (the cornfield diet), which is a plant-based diet composed of corn, beans, zucchini, and chili, and also includes a large number of native fresh fruits and vegetables, like citrus, papaya, quelites, red tomato, and cactus (nopales). It also comprises protein-rich seeds such as pumpkin seeds, chia, amaranth, and peanuts, and healthy fats like avocado. Fermented drinks, homemade cheese, and insects are also present in that diet (Lares-Michel et al., 2022; Valerino-Perea et al., 2019).

The sustainability of the Mexican traditional diet has been recently claimed and proposed as an alternative for promoting human and planetary health in specific regions like Mexico, meanwhile promoting cultural and affordable diets (Almaguer González et al., 2019; Lares-Michel et al., 2022; 2023). Nevertheless, the principal problem regarding recovering traditional diets is to ensure that the population adheres to these kinds of dietary patterns. Currently, it has been reported that the Mexican population is consuming a Western diet with high environmental implications and detrimental to their health (Aburto et al., 2022; Batis et al., 2021; López-Olmedo et al., 2022).

Regarding gut microbiota, a study in mice showed that a traditional Mexican diet based on corn, beans, tomato, nopal, chia, and pumpkin seeds in a dehydrated form significantly increases the relative abundances of *Akkermansia* and *Bifidobacterium.* Also, its consumption decreased glucose intolerance and the biochemical abnormalities caused by obesity by increasing the abundance of fatty acid oxidation proteins and decreasing oxidative stress (Avila-Nava et al., 2017).

Despite their beneficial effects on gut microbiota and the environment have been suggested, it is essential to mention that the personalisation of these dietary patterns is needed (Grasso et al., 2022). Also, the specific impact of this diet on environmental indicators, such as the water and carbon footprints and health outcomes, including the gut microbiota, needs to be known to promote the diet accurately. Besides, due to nutrition transition, behavioural interventions are needed to increase adherence to traditional diets, respecting sustainable diet dimensions (Biasini et al., 2021; Vos et al., 2022).

Fortunately, currently an RCT is being performed in Mexico, where a diet is being mathematically optimised to design a healthy, sustainable diet for Mexico’s context based on its traditional diet (Lares-Michel et al., 2023). The study will report the effects of this diet on specific bacteria such as Bacillota, Bacteroidota, *Lactobacillus*, *Bifidobacterium*, *F. prausnitzii*, *A. muciniphila*, *P. copri*, *B. wadsworthia*, *Clostridium coccoides*, and *Streptococcus thermophilus.* Besides, it includes environmental indicators and will cover all sustainable diet dimensions (Lares-Michel et al., 2023). When this research’s results are available, the gut microbiota effects of a personalised region-specific sustainable diet could be characterised for the first time.

## Sustainable diet’s composition and its potential effects on the gut microbiome

Although healthy and sustainable diets used to be addressed separately, in 2021, the United Nations Food Systems Summit proposed a new definition for a healthy diet, referring to a healthy diet such as one that is human health-promoting and disease-preventing, and safeguarding of planetary health by providing adequacy, without excess, of nutrients from foods that are nutritious and healthy, avoiding the introduction of health-harming substances, through all stages of the value chain. Healthy diets must be affordable and culturally acceptable. They must progressively change towards originating from sustainable production and processing systems that do not adversely affect local and regional ecologies (Neufeld et al., 2023). In this regard, the most up-to-date literature about healthy and sustainable diets starts addressing them as concepts that cannot be separated.

Currently, the most accepted definition of sustainable diets is the one proposed by the Food and Agriculture Organization of the United Nations and World Health Organization (2019), which defines this kind of diet as dietary patterns that promote all the dimensions of people’s health and well-being, meanwhile having low pressure on the environment, and being accessible, affordable, safe, and equitable for all populations, respecting the culture of the people. Nevertheless, the principal problem with these diets is that to achieve a person’s adherence to a diet of this type, many behaviours must be addressed simultaneously to modify current dietary habits. However, simultaneously ensuring the well-being of the population’s environment, health, nutrition, culture, and socioeconomic aspects is a big challenge (Biasini et al., 2021; Drewnowski, 2020).

One of the most recognised diets for sustainability is the Planetary Health Diet, created in 2019 by researchers from the EAT-Lancet Commission. This diet model provided specific recommendations to promote healthy diets derived from sustainable food systems (Rehner et al., 2023). However, this dietary pattern has been criticised in several ways (Verkerk, 2019). First, it was criticised for not considering specific regions’ cultural elements, and its affordability has been questioned (Drewnowski, 2020). Besides, its macro and micronutrient profiles were pointed out as lacking adequacy, especially considering those generally found in higher quantities and more bioavailable forms in animal-source foods (Beal et al., 2023). Also, its practical implementation feasibility has been repeatedly referred to as one of the principal limitations of this diet (Breidenassel et al., 2022).

Despite the limitations of the Planetary Health Diet, a recent study reported its effects on the Human Gut Microbiome (Rehner et al., 2023). The study was conducted on 41 German adults (22–57 years). The sample was divided into three groups, one consuming a Western omnivorous diet, the second consuming a vegan or vegetarian diet, and the third consuming the EAT-Lancet diet for Planetary Health for 12 weeks. In this study, a trend towards an increase in *Bifidobacterium adolescentis* and *Coprococcus eutactus* was identified in the group consuming the Planetary Health Diet (Rehner et al., 2023). *B. adolescentis* is capable of degrading inulin (a type of fibre) into lactate and acetate, which can be used by *Anaerostipes hadrus* and *Enterococcus rectale* to produce the SCFA butyrate, thus generating anti-inflammatory effects on the host (Baxter et al., 2019). Another bacteria found after consuming the Planetary Health Diet was *P. copri*, which, although has been related to some beneficial metabolic effects, such as glucose regulation (Asnicar et al., 2021; Losno et al., 2021), has also been related to the development of rheumatoid arthritis (Scher et al., 2013). However, more studies are needed since this bacteria has been highly associated with healthy diets, especially plant-based (Losno et al., 2021; Trefflich et al., 2020).

Despite the exciting insights that these emerging studies are providing to the study of the gut microbiota composition of sustainable diets, the sustainability limitations addressed before provide direction for future studies that characterise the composition of the gut microbiota generated by the consumption of sustainable, contextualised, and personalised diets, covering all sustainability dimensions. To the best of our knowledge, no study has analysed the effects that a sustainable diet covering all those elements could have on the gut microbiome.

PubMed database reports zero research when using the terms (“gut microbiota” OR “gut microbiome” OR “gut bacteria” OR “microbiota diversity” OR “intestinal bacteria” OR “gut bacteria” OR “intestinal flora”) AND (“sustainable diet”). Only three registered protocols on ClinicalTrials.com are in course for assessing the effects of sustainable diets on gut microbiota. The first protocol is being carried out in the United Kingdom (ClinicalTrials.gov Identifier: NCT05231317). To the moment, the authors have included 20 participants (18–70 years) that were divided into an experimental plant-based diet group, which was referred to as a diet rich in fruit and vegetables (42% carbohydrates, 17.2% fibres; 15% proteins; and 43% fats), during 16 days with all foods provided. Furthermore, an active comparator group following a Western diet based on processed foods (48% carbohydrates, 10.4% fibres; 14% proteins; and 39% fats) was included. The study’s primary outcomes are significant changes in the number of *Bifidobacteria* over 14 days and changes in Trimethylamine N-oxide over 16 days assessed by changes in response to L-Carnitine challenge (ClinicalTrials.gov, 2022a). Although this study is one of the pioneers in the study of the gut microbiome of sustainable diets, it has been noted that the inclusion of the sustainability diet elements is not mentioned, and besides, no specific behavioural techniques are detailed for guaranteeing the population actually adheres to the prescribed diet (ClinicalTrials.gov, 2022a).

The second study is being developed in Mexico and is based on a Sustainable-psycho-nutritional Intervention Program that aims to promote adherence to a region-specific sustainable diet and to evaluate their effects on several environmental and health outcomes, including the gut microbiota (ClinicalTrials.gov Identifier: NCT05457439). The study is an RCT, targeting a control (n = 50) and intervention (n = 50) group (18–35 years), with metabolic-health risk factors. The intervention group followed a sustainable diet mathematically designed for Mexico’s context for 7 weeks. This was then followed up for 7 more weeks. The control group did not receive any intervention or nutritional advice. Regarding gut microbiome, the study evaluates the changes in relative abundances of specific bacteria related to metabolic health or dietary patterns, such as *Bacillota*, Bacteroidota, *Lactobacillus*, *Bifidobacterium*, *F. prausnitzii*, *A. muciniphila*, *P. copri*, *B. wadsworthia*, *C. coccoides*, and *S. thermophilus* (ClinicalTrials.gov, 2022b). No results have been reported, but the current Clinical Trials report indicates the project is about to get completed.

Finally, the most recent registered study on Clinical Trials is another study in Mexico that aims to Reduce Metabolic Endotoxemia in 100 adults through a low-inflammatory and environmentally friendly dietary strategy (ClinicalTrials.gov Identifier: NCT05776329). Among their primary outcomes is a change in Lipopolysaccharide-binding protein, and secondarily the study will evaluate *Prevotella* changes. All variables are measured over 6 weeks (ClinicalTrials.gov, 2023). Also, no results are yet available.

Although evidence is still scarce for defining the composition of gut microbiota related to sustainable diet consumption, based on available data, it can be assumed that sustainable diets could promote the proliferation of the same or at least similar bacteria observed in plant-based diets (Rehner et al., 2023). However, it must be remembered that sustainable diets are not necessarily vegan or vegetarian and can include minor or even moderate amounts of animal-based foods, especially considering that for a diet to be sustainable, personal food preferences, culture, and societal aspects of each individual must be addressed (Biasini et al., 2021; Carey et al., 2023). Also, exploring how locally produced food can affect individuals in specific regions is exciting. Some previous studies have shown that geographical location is a crucial determinant of gut microbiota, so this must also be considered when exploring the sustainability context (Senghor et al., 2018).

Those variations among diets, geographical zones, and gut microbiota composition and functions could also confirm the role of bacteria on human evolution and how bacteria can play a role in human adaptation to specific contexts, climates, soil types, and of course, food intake. However, not only could bacteria have a role in this process, but diet could modulate the differences in the gut microbiota of ancestors in contrast with modern societies (Elechi et al., 2023). Also, the evolution of the food system, and especially the use of herbicides and fertilisers, could have influenced the gut microbiota of individuals. Seasonal food consumption is also a characteristic of sustainable diets, for which specific environmental characteristics such as climate could also impact gut microbiota (Elechi et al., 2023; Robinson et al., 2018). Therefore, returning to consuming a diet based on un-processed and vegetable foods, locally produced in natural seasons and produced respecting the environment, is promising for a gut microbiota aligned with health (González Olmo et al., 2021).

Exploring the gut microbiota and its metabolites in relation to sustainable diets should be further studied to allow the configuration of even more personalised nutrition for gut microbiota modulation. Nowadays, we know that more diverse gut microbiota and a higher proliferation of butyrate-producing bacteria, considered anti-inflammatory, are essential for health preservation. So far, the scientific consensus points towards rich fibre diets, low in ultra-processed foods, and animal origin to improve human intestinal microbiota’s diversity and population and preserve its proper functioning (McCarthy and Li, 2019; Rinninella et al., 2019). Even the consumption of beef (the food with the most significant environmental impact) has been related to a decrease in beneficial bacteria such as *Bifidobacterium*, and the substitution of animal protein for vegetable sources has been referred to as a suitable option to increase the number of health-related bacteria in the body (*Bifidobacterium* and *Lactobacillus*) and reduce those that are considered pathogenic (*B. fragilis* and *Clostridium perfringens*) (Świątecka et al., 2011).

Some insight has emerged for specific regional dietary patterns, such as the Mediterranean and the Mexican traditional or milpa, suggesting that their current composition, which is principally plant-based, could be sustainable and could improve gut microbiota composition (Avila-Nava et al., 2017; Muralidharan et al., 2021). However, no study has addressed all sustainability dimensions of those dietary patterns in relation to the gut microbiota composition and functions of the population consuming those diets. Also, the composition of the diet for planetary health of the EAT-Lancet Commission, which is also a plant-based diet, is promising for the gut microbiota, considering fibre content, antioxidants, and vegetable protein. Nevertheless, as far as no contextualisation to specific regions is done, that diet cannot be considered sustainable (Rehner et al., 2023).

Therefore, so far, the characteristics of a diet that benefits the intestinal microbiota coincide with the elements of a diet that optimises the use of natural resources and generates low environmental impacts. However, in-depth studies identifying production regions, and their related characteristics (soil characteristics, climate, food production techniques, use of herbicides), could bring new perspectives and further characterise what a sustainable diet gut microbiota composition and functions are (Biasini et al., 2021; Rehner et al., 2023). Also, it is essential to recognise that evolution has not stopped, and technologies will keep emerging. Therefore, they should be used to improve current food production and direct it towards more sustainable techniques. Even technologies can help produce sustainable, functional food ingredients to modulate the human gut microbiota (de Carvalho et al., 2023).

## Conclusions

Characterising the gut microbiome of individuals consuming healthy sustainable diets is essential to generate evidence of their effects on the planet and the population’s health status. Modulating the gut microbiome could regulate health status, and its role in sustainable food production and biodiversity preservation must arise. It is urgent to stop generalising and assuming healthy or plant-based diets to be sustainable because contextualisation and personalisation are necessary for a dietary pattern to be considered sustainable. Thus, consistently, for a diet to be sustainable, it must consider health and nutrition, environment, cultural and socioeconomic aspects. Finally, multidisciplinary behavioural interventions and public health policies are needed to encourage healthy behaviour and change current food environments to achieve the potential benefits of sustainable diets on gut microbiota. It is also important to consider strategies to reduce food waste, which will significantly benefit the environment, and to reduce caloric intake, which will benefit both the environment and human health. Precision nutrition will likely play a role in optimising personal health in the future. However, much more research is needed before it can be widely applied to disease prevention and planet conservation while improving health outcomes such as the gut microbiome (Hemler and Hu, 2019).
